# VGLUT2 controls heat and punctuate hyperalgesia associated with nerve injury via TRPV1-Cre primary afferents

**DOI:** 10.1371/journal.pone.0116568

**Published:** 2015-01-23

**Authors:** Katarzyna Rogoz, Ludvig Stjärne, Klas Kullander, Malin C. Lagerström

**Affiliations:** Department of Neuroscience, Uppsala University, Uppsala, Sweden; University of South California, UNITED STATES

## Abstract

Nerve injury induces a state of prolonged thermal and mechanical hypersensitivity in the innervated area, causing distress in affected individuals. Nerve injury-induced hypersensitivity is partially due to increased activity and thereby sustained release of neurotransmitters from the injured fibers. Glutamate, a prominent neurotransmitter in primary afferents, plays a major role in development of hypersensitivity. Glutamate is packed in vesicles by vesicular glutamate transporters (VGLUTs) to enable controlled release upon depolarization. While a role for peripheral VGLUTs in nerve injury-induced pain is established, their contribution in specific peripheral neuronal populations is unresolved. We investigated the role of VGLUT2, expressed by transient receptor potential vanilloid (TRPV1) fibers, in nerve injury-induced hypersensitivity. Our data shows that removal of *Vglut2* from Trpv1-Cre neurons using transgenic mice abolished both heat and punctuate hyperalgesia associated with nerve injury. In contrast, the development of cold hypersensitivity after nerve injury was unaltered. Here, we show that, VGLUT2-mediated glutamatergic transmission from Trpv1-Cre neurons selectively mediates heat and mechanical hypersensitivity associated with nerve injury. Our data clarifies the role of the Trpv1-Cre population and the dependence of VGLUT2-mediated glutamatergic transmission in nerve injury-induced hyperalgesia.

## Introduction

Hyperalgesia and allodynia are two forms of hypersensitivity that depend on peripheral as well as central alterations of sensory transmission following injury. A candidate for peripheral nociceptor sensitization is the cation-selective ion-channel TRPV1 (transient receptor potential vanilloid 1) [1, 2, 3]. The role of TRPV1 in hyperalgesia has been studied in various models where the contribution of TRPV1 to heat hyperalgesia during inflammatory states is well established. Mice lacking TRPV1 are characterized by attenuated development of heat hyperalgesia during tissue inflammation [1], similarly, genetic deletion of the Trpv1-Cre population [3] leads to decreased heat hypersensitivity during inflammatory states. However, the specific transmission accounting for the development of different modalities of nerve injury-induced hypersensitivity involving TRPV1 neurons remains unknown.

Initial reports have shown that TRPV1 is ubiquitous for the mediation of heat or punctuate hyperalgesia after nerve injury, suggesting that TRPV1 is only critical for hyperalgesia after certain types of tissue injury, excluding nerve damage [1]. However, in models of neuropathic pain, expression levels of TRPV1 in undamaged neurons increase, whereas in damaged neurons they decrease [4]. Also, peri-sciatic administration of capsaicin and QX-314 reduces both heat and mechanical hypersensitivity in the chronic constriction injury model [5], indicating that the TRPV1 receptor and population might be involved in the development of neuropathic pain. To define the role of the TRPV1 population in nerve injury, we here set out to further characterize the population and the neurotransmitters involved in the transmission via these primary afferents.

A role for spinal glutamate in the development of chronic pain has been previously suggested. Pharmacological or antisense manipulation of glutamate receptors leads to reduced punctuate hyperalgesia following peripheral nerve injury [6, 7] and decreased reuptake of glutamate via spinal glutamate transporters can contribute to pathogenesis in different neuropathic pain models [8, 9]. It is currently unclear to which extent peripherally delivered glutamate contributes to these processes. Introduction of genetic tools, like conditional deletion of vesicular glutamate transporters (VGLUTs), have provided an approach to silence glutamatergic signaling in defined primary afferent populations [10, 11, 12, 13, 14]. The use of such mice has resulted in an increased knowledge of glutamate-mediated neurotransmission from peripheral neurons in different states of hypersensitivity. We have previously shown that VGLUT2-mediated transmission from all primary afferents is crucial for the development of heat, cold and punctuate hyperalgesia [15]. By manipulating more specific neuronal populations, distinct roles of VGLUT2 and the population in question can be defined.

Here, we have used a genetic approach to specifically delete Trpv1-Cre expressing neurons, or their expression of VGLUT2, to investigate the contribution from Trpv1-Cre neurons to the development of different modalities of nerve injury-associated hypersensitivity.

## Materials and Methods

### Generation of transgenic animals

Mice heterozygous for the for diphtheria toxin allele Gt(ROSA)26Sor^tm1(DTA)Jpmb^, (129/SvEv * C57BL6/J) hereafter called *R26* ^DTA^ [16] were crossed with mice heterozygous for the Trpv1-Cre allele (C57BL/6NCrl*DBA/2) [10] to generate mice lacking Trpv1-Cre positive neurons (*R26* ^DTA^; Trpv1-Cre^tg/wt^) and controls (*R26* ^DTA^; Trpv1-Cre^wt/wt^ or *R26* ^wt/*wt*^; Trpv1-Cre^wt/wt^). The Trpv1-Cre mice were also crossed with *Vglut2* ^f/f^ mice (Sv129/R1 * C57BL/6) [17] to generate mice where *Vglut2* was selectively removed from Trpv1-Cre expressing neurons (*Vglut2* ^f/f^;Trpv1-Cre). For analyses of the expression of Trpv1-Cre, crossings between Trpv1-Cre line and the reporter line tdTomato (Gt(ROSA)26Sor^tm14(CAG-tdTomato)Hze^; Allen Brain Institute) were done.

### Genotyping by PCR

First, 1–2 mm tail was incubated in 75 μl of buffer consisting of 25 mM NaOH and 200 μM EDTA at 96°C for 45 min and placed on ice before the sample was neutralized with 75 μl of Tris-HCl (40 mM) pH 8.0. Mice were genotyped for the presence of the Trpv1-Cre, the R26^DTA^ allele, the *Vglut2* ^f/f^ allele and tdTomato allele. Mice were analyzed using the following primers: Trpv1-Cre: 5´- GTGCAAGCTGAACAACAGGA (forward), 5´- CCAGCATCCACATTCTCCTT (reverse). *R26* ^DTA^: 5´- GTTATCAGTAAGGGAGCTGCAGTGG, 5´- AAGACCGCGAAGAGTTTGTCCTC, 5´- GGCGGATCACAAGCAATAATAACC. *Vglut2* ^f/f^: 5´-CTGTCCACCTTTGTATCCCA (forward), 5´- GCAATCACATTTCACTGTTC (reverse, floxed allele) and 5´- CACACCCACTCCACTTGAGG (reverse, excised allele). *tdTomato*: 5´- CTGTTCCTGTACGGCATGG (forward), 5´- GGCATTAAAGCAGCGTATCC (reverse), 5´-AAGGGAGCTGCAGTGGAGTA (forward), 5´- CCGAAAATCTGTGGGAAGTC (reverse).

### Multiplex single-cell PCR

Dorsal root ganglia were dissected from Trpv1-Cre mice and collected in ice-cold Leibovitz’s L-15 Medium (L15) (Invitrogen). The tissue was treated with dispase (Sigma) for 30 min at 37°C and then with trypsin (Invitrogen) for 15 min at 37°C. Trypsin was neutralized and the tissue was triturated by several passages through glass pipettes of decreasing diameter to obtain a cell suspension, and subsequently gradient centrifuged to eliminate dead cells and debris. Cells were plated on poly-L-lysine-coated coverslips and left to adhere for 30 min at 37°C and then washed with Krebs-Ringer buffer (KRB) (in mM: 140 NaCl, 5 KCl, 2 MgCl_2_, 2 CaCl_2_, 10 HEPES, 10 glucose, 6 sucrose, pH 7.35) to eliminate non-attached cells. Coverslips were kept in KRB during single cell collection. Cells were individually collected under RNAse-free conditions using autoclaved borosilicate patch pipettes; each cell was collected by applying light negative pressure to the pipette, no intracellular pipette solution was used. The content of each pipette was transferred immediately into individual pre-chilled 200 μl tubes containing 6μl of a freshly prepared solution of 20 U of RNaseOUT (Fermentas) and 8.3 mM DTT (Invitrogen), samples were immediately frozen on dry ice until use. Frozen samples were thawed on ice and subjected to cDNA synthesis for 1h using 0.5 mM dNTPs mix, 1.25 μM random hexamers (Invitrogen), 40 U of RNase inhibitor (Fermentas), 100 U of M-MLV RT (Invitrogen), 50 mM Tris-HCl, 75 mM KCl and 3 mM MgCl_2_, pH 8.3. The RT enzyme was denatured and the cDNAs stored at -80°C until use. A first round of PCR was performed using 1.5 mM MgCl_2_, 10 pmol of each primer, 1.0 U of Maxima Hot Start Taq Polymerase (Fermentas), 20 mM Tris-HCl and 10 mM KCl pH 8.3 and 35 cycles at 55°C annealing temperature. A second round of PCR was performed using 10% of the first PCR reaction. Primers were designed based upon sequences deposited in the GenBank database (www.ncbi.nlm.nih.gov/nucleotide). They were designed to bind different exons, therefore they detect only mRNA and not genomic DNA. Ladder: 100 bp (Fermentas). Right primers are followed by left primers: *Cre* 5’-ggaagatgctcctgtctgtg-3’ and 5’-gatttcagggatggacacac-3’; *Cre* nested 5’-tgaggatgtgagggactacc-3’ and 5’-ttctccatcagggatctgac-3’, *MrgprD* 5´- gagaagggagaggctaccag-3’ and 5’-tgcaccagataccactgatg-3’; *MrgprD nested:* 5’-gaaggacagattggcacact -3’ and 5’-ggccatgcagaataagaaga-3’.

### Tissue preparation

Mice (>7 weeks old) were perfused as (previously described [18]. Dorsal root ganglia (DRG) and spinal cord from lumbar and sacral segments were isolated for *in situ* hybridization and immunohistochemistry. The isolated tissue was fixed in fresh 4% PFA for 2 hours shaking on ice followed by a graded series of sucrose solutions ending at 30% sucrose, before the tissue was mounted and frozen in O.C.T compound (Sakura Finetek, Zoeterwounde, NL) at -80°C. Sections of 14 µm were cut and mounted on Superfrost glass (Menzel-Gläser, Braunschweig, Germany) and stored at -80°C. For developmental studies, the female underwent cervical dislocation prior to all procedures and embryos at chosen stages: E12.5, E13.5 and E15.5 were dissected out from the uterus. Embryos were placed in an isofluran chamber, until all reflexes were gone, and then washed in 4% PFA, followed by gradient of sucrose. Subsequent procedures were equivalent to those performed on adult tissue, with the difference that embryo tissue was cut coronally.

### Immunohistochemistry

Sections were briefly rinsed in 1xTBS, followed by an incubation in 10% methanol and 3–4% H_2_O_2_ in 1xTBS, before the sections were rinsed in 1xTBS and incubated overnight in 4°C in blocking buffer with 0.5% gelatin and 0.01% Triton-X in TBS supplied the primary antibody for anti β-tubulin 1:500 rabbit (Biosite) or anti red fluorescent protein (RFP) rabbit 1:200 (Abcam). The sections were then rinsed repeatedly in 1xTBS followed by incubation for 1 h in RT in blocking buffer with 0.5% gelatin and 0.01% Triton-X in TBS supplied with secondary antibody goat anti-rabbit Alexa 594 1:800 (Invitrogen) or anti-rabbit Alexa 647 1:400 (Invitrogen).

### In situ hybridization and immunohistochemistry

The sections were rinsed repeatedly in 1xPBS followed by a pre-block in 0.3% BSA, 0.1% Triton-X in 1xPBS for 30 min and incubated overnight in 4°C blocking buffer supplied with the primary antibody for anti-β-galactosidase (β-gal) 1:5000 chicken (Abcam). The sections were then rinsed repeatedly in 1xPBS, followed by incubation for 1 h in RT in blocking buffer supplied with the secondary antibody goat anti-chicken Alexa 488 1:400 (Invitrogen). The sections were washed with 1xPBS before the cryo *in situ* hybridization commenced. Cryo *in situ* was performed as described previously [19] with a few exceptions. Briefly, sections were fixed in 4% PFA, followed by repeated washes in 1xPBS, incubation with Proteinase K in 10 mM Tris-HCl, pH 8.0 for 8 min followed by washes with 1xPBS and acetylation for 10 min. Sections were then briefly rinsed before incubation in hybridization buffer (50% formamide, 5xSSC, 5xDenharts, 500µg/ml salmon sperm DNA, 50µg/ml tRNA) in 55°C for 1 h. Sections were then incubated with hybridization buffer supplied with the denaturated *Vglut2* probe in 55°C for 6 h. Sections were then briefly washed with 5xSSC and later 0.2xSSC for 1 h at 55°C before equilibration in 1xTBS at RT. The pre-block was performed at RT for 1 h in 1xTBS supplied with 1xBlocking reagent (Roche) before the sections were incubated with blocking buffer containing anti-digoxigenin AP Fab fragments 1:5000 (Roche) and left overnight in 4°C. Sections were later washed with 1xTBS supplied with 0.1% Tween-20 and 2mM Levamisole (Sigma). Development was performed at 37°C using filtrated Fast Red (Roche) 0.1nM Tris HCl, pH8.2 solution. When the development was finished, the sections were again incubated with the secondary antibodies used prior to the *in situ* hybridization to strengthen the signal. Sections were then washed and closed with cover slips.

### Imaging

Fluorescent images were viewed in an Olympus BX61WI microscope (Olympus, Sweden) and analyzed using the Volocity software (Improvision, Lexington, USA). The Mirax software (Carl Zeiss MicroImaging Gmbh) was used for embryos analyzes.

### General behavior

All behavioral tests were performed on adult (>7 weeks old) female and male mice. Control mice were littermates and gender matched. All behavior analyses were performed in a controlled environment of 20–24°C, 45–65% humidity and 12 h day/night cycle.

### Ethics statement

All animal procedures were approved by the local ethical committee (Uppsala djurförsöksetiska nämnd) at Uppsala tingsrätt, following the Directive 2010/63/EU of the European Parliament and of the Council, The Swedish Animal Welfare Act (Djurskyddslagen: SFS 1988:534), The Swedish Animal Welfare Ordinance (Djurskyddsförordningen: SFS 1988:539) and the provisions regarding the use of animals for scientific purposes: DFS 2004:15 and SJVFS 2012:26. All behavior experiments were performed by an observer blind to the genotype.

### Hargreaves test

The mice were acclimatized in transparent plexiglass chambers with a glass floor for 30–60 min or until no exploratory behavior was observed. The Hargreaves heat source (IITC Life Science, Woodland Hills, CA, USA) was placed with the guide light pointing towards the plantar surface of a hind paw and the thermal beam was started. A paw withdrawal would stop the test and the time was monitored. The cut off time was set to 20 s. The test was repeated at least three times/animal allowing at least 5 min in between each test. The result was expressed as the mean withdrawal latency time for each animal and group ± SEM.

### von Frey test

The mice were acclimatized in elevated transparent chambers with a metal mesh floor for 60–90 min or until any exploratory behavior had stopped. The von Frey filaments (Scientific Marketing Associates, UK) were applied using the Chaplan “up and down paradigm” [20]. Response to a hair filament would be the withdrawal of the paw from the applied hair. The lack of response to the application of one hair-filament would lead to the application of a thicker hair. Likewise, a response, would lead to the next application being of a thinner size. The experiment would stop when six responses had been monitored around the 50% threshold. All experiments were initiated using filament 0.4g. The 50% threshold was calculated using DIXON method [21] and the result expressed as the mean value for each animal and group ± SEM.

### Acetone test

The mice were acclimatized in elevated transparent chambers with a metal mesh floor for 60–90 min or until any exploratory behaviour had stopped. Acetone diluted with water 9:1 was applied as a droplet to the plantar surface of the left hind paw of the animal using a one ml syringe. The drop but not the syringe touched the paw from below and the time the mouse showed signs of pain, i.e. lifting, flinching and licking was measured with a timer during one minute. The test was repeated three times with at least five minutes in between. The result was expressed as the mean withdrawal latency time for each animal and group ± SEM.

### Partial Sciatic Nerve Ligation (PSNL)

To introduce nerve injury, animals were anaesthetized using approximately 2% isoflurane mixed with oxygen (29%) and nitrogen (69%). An incision was made in the skin parallel to the left hind limb and a further separation of the underlying muscles was made to access the sciatic nerve [22]. 1/3–1/2 of the sciatic nerve was ligated with 6–0 Ethilon suture (Agnthos) before the skin was ligated using a 4–0 Vicryl suture (Agnthos). Sham-operated animals were treated in the same way, but without ligation of the sciatic nerve. Baseline measurements for thermal (acetone (cold allodynia)) and Hargreaves test (heat hyperalgesia)) and mechanical (von Frey (punctuate hyperalgesia)) responses were measured -3-(-1) days prior to the surgery (day 0). The acetone test and von Frey test were performed on day 3, 5, 7, 10, 15, 20, 25 and 30 post surgery. Hargreaves test was performed on day 6, 11, 16, 21, 26 and 31 post surgery. The experimenters were blind to the genotype and type of surgery performed (sham or PSNL). Animals were sacrificed and re-opened after the last measurement to ensure intact ligation. The results were presented both as mean response/group ± SEM and mean response/animal pre injury vs. mean response/animal post injury ± SEM.

### Statistics

Non-parametric calculations of p-values were conducted using Mann-Whitney two-tailed test (Prism version 5.01, GraphPad Software Inc, CA, USA). For negative results the power of the test was calculated based on evaluating type II error.

## Results

### The Trpv1-Cre population of primary afferents overlaps with VGLUT2

**Figure 1:**
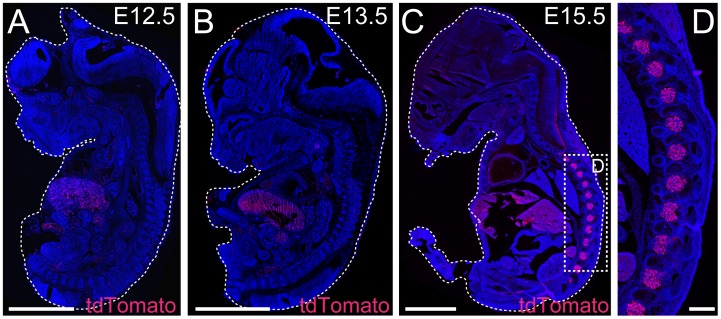
Trpv1-Cre show prominent activity at E15.5. The onset of Trpv1-Cre expression was determined using the reporter line tdTomato. **(A-D)** Whole-embryo micrographs of Trpv1-Cre;tdTomato at the different developmental stages; E12.5, E13.5 and E15.5. No tdTomato expression was observed at E12.5, a few DRG cells expressed Trpv1-Cre at E13.5 and profound expression of tdTomato was observed at E15.5 in DRGs. Scale bars = 2000μm (A-B), 2500μm (C), 500μm (D). Auto fluorescence was observed in abdominal tissue (see S1 Fig. for details).

**Figure 2:**
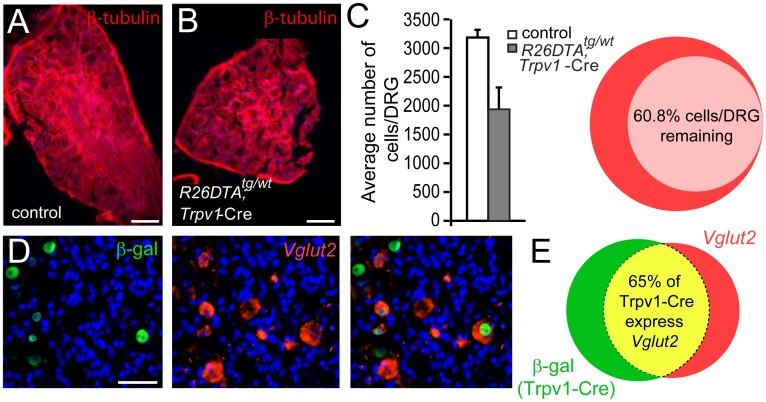
The Trpv1-Cre population co-express *Vglut2* to a great extent. **(A-B)** The Trpv1-Cre population constitutes 39.2% of the primary afferent neurons (n = 3/genotype, 1 DRG per animal, 77 sections/ *Vglut2* ^f/f^;Trpv1-Cre mice and 95 sections/ctrl). **(C-D)** Trpv1-Cre neurons overlap greatly with *Vglut2* mRNA, 65.1±2.2% of Trpv1-Cre neurons express *Vglut2* which corresponds to 73.8±2.1% of the *Vglut2* population (n = 3, 35 number of sections). Scale bar = 33 μm.

To characterize the primary afferent TRPV1 population and further define its role in hyperalgesia following peripheral nerve injury, we used Trpv1-Cre mice in combination with the reporter line tdTomato and the diphtheria toxin line *R26* ^DTA^ to visualize and delete the neurons, respectively. We have previously shown that the Trpv1-Cre line display prominent expression of Cre recombinase in TRPV1 expressing primary afferent neurons (93% of TRPV1 neurons overlap with Trpv1-Cre) and very limited expression in the central nervous system [10]. However, besides targeting almost all TRPV1 immunoreactive neurons, it should be clarified that the TRPV1 promoter is also active in a population of primary afferents during development which in adult does not express TRPV1 [3, 23, 24]. We therefore refer to the population studied here as Trpv1-Cre neurons. In Trpv1-Cre mice, a small number of Cre active cells could be detected at E13.5 whereas the activity was prominent from embryonic stage E15.5, determined using the tdTomato reporter line (Fig. 1A-D). We have previously shown that deletion of the Trpv1-Cre population, using the *R26^DTA^* line, removes 99.1% of all Trpv1-Cre cells [25] and in total 39.2% of primary afferent neurons per DRG in *R26* ^DTA^; Trpv1-Cre^tg/wt^ compared to control littermates were lost. The number of cells was reduced from 3184±134/DRG present in control litter mates to 1937±375/DRG in *R26* ^DTA^; Trpv1-Cre^tg/wt^ mice (Fig. 2A-C).

The Trpv1-Cre population is a heterogeneous population based on size distribution and transmitter content and has previously been shown to express markers for glutamatergic [10] as well as for peptidergic signaling [13, 23]. We further confirmed the overlap between the Trpv1-Cre population and glutamate by analyzing the expression of *Vglut2* mRNA in Trpv1-Cre cells which demonstrated that a majority, 65.1±2.2%, of the Trpv1-Cre population expresses *Vglut2* mRNA (Fig. 2D-E). This is in agreement with our previous overlap analysis of *Vglut2* and Trpv1-Cre [10, 13, 25].

### VGLUT2-mediated glutamatergic transmission in Trpv1-Cre neurons is responsible for heat, but not cold, hyperalgesia following nerve injury

**Figure 3:**
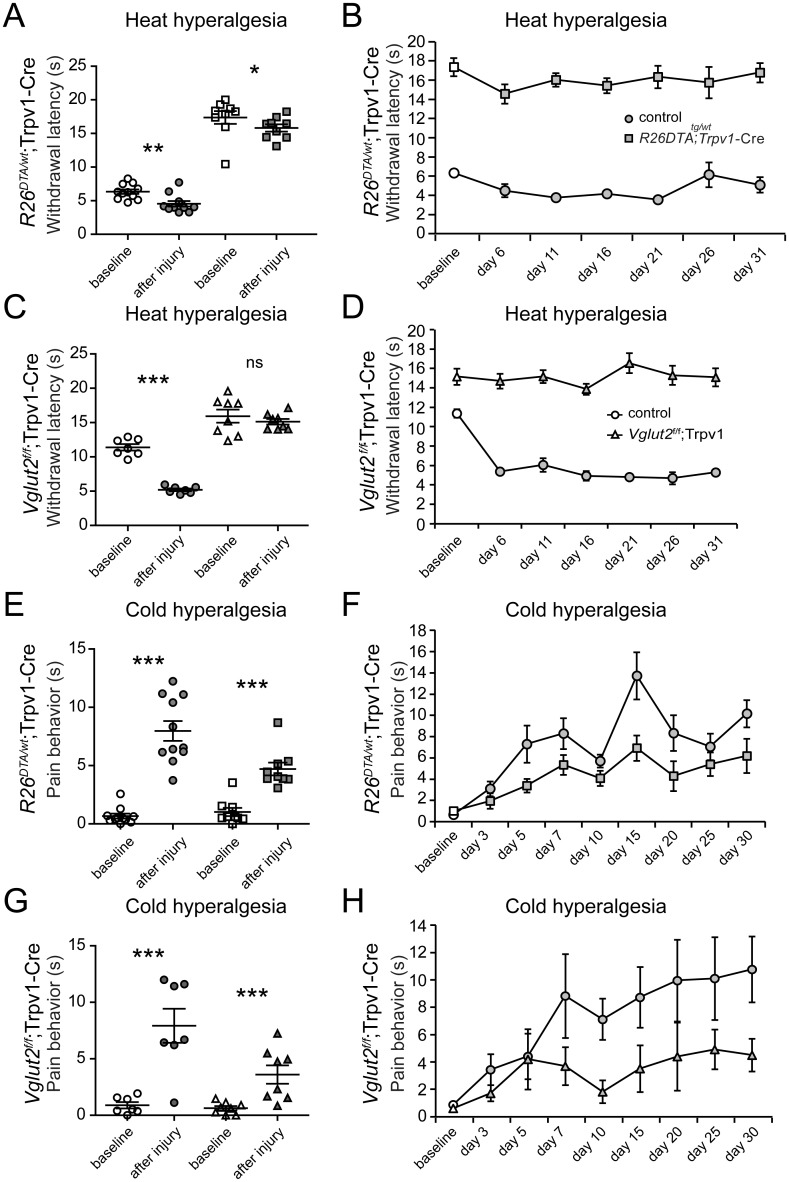
Glutamate mediates heat, but not cold, hyperalgesia associated with peripheral nerve injury via the Trpv1-Cre population. **(A-B)** Both control (n = 11) and *R26* ^DTA/w*t*^;Trpv1-Cre (n = 9) mice develop heat hyperalgesia but transgenes to a lesser extent. **(C-D)** Control (n = 7), but not *Vglut2* ^f/f^;Trpv1-Cre mice (n = 8), develop heat hyperalgesia following PSNL. (E-F) Both control (n = 11) and *R26* ^DTA/w*t*^;Trpv1-Cre (n = 9) mice develop cold hyperalgesia. **(G-H)** Similarly, both controls and *Vglut2* ^f/f^;Trpv1-Cre mice develop cold hyperalgesia. Baseline (white) = response before PSNL, after injury (grey) = average response after PSNL. Circles denotes controls, squares display *R26* ^DTA/w*t*^;Trpv1-Cre and triangles show *Vglut2* ^f/f^;Trpv1-Cre mice. * p<0.05, **p<0.01, *** p<0.001, ns p>0.05, Mann-Whitney two-tailed test.

The significant overlap of the Trpv1-Cre population with *Vglut2* suggests a possible contribution of glutamatergic transmission to Trpv1-Cre neuronal signaling. Therefore, we cross-bred the Trpv1-Cre line with the *Vglut2*-lox line [17] to determine the role of VGLUT2-mediated transmission in Trpv1-Cre neurons in hyperalgesia associated with nerve injury. These results were compared with the analysis of mice lacking the Trpv1-Cre population using the diphtheria toxin line. TRPV1 has been shown to play a critical role for the development of heat hyperalgesia associated with inflammation in mice lacking TRPV1 [1] or the Trpv1-Cre population [3, 13] but the role of the Trpv1-Cre population in heat hyperalgesia after nerve injury is not defined. Our analysis showed that a state of heat hyperalgesia developed both in control PSNL mice and in *R26* ^DTA/w*t*^; Trpv1-Cre PSNL mice; 6.3±0.3 s vs. 4.5±0.4 s (pre injury vs. mean post injury; p = 0.006) vs. 17.4±0.9 s vs. 15.8±0.5 s (p = 0.04) (Fig. 3A-B). However, *R26* ^DTA/w*t*^; Trpv1-Cre PSNL mice developed a less pronounced heat hyperalgesia compared to control PSNL mice (p = 0.04), indicating that Trpv1-Cre neurons contribute to the development also of heat hyperalgesia associated with nerve injury.

Removal of VGLUT2 from all primary afferents results in attenuated or absent thermal hyperalgesia following nerve injury [15], thus, we wished to analyze the contribution from the Trpv1-Cre population of primary afferents. In our experimental set up, control mice developed heat hyperalgesia; 11.2±0.4 s vs. 5.1±0.2 s (pre injury vs. mean post injury; p = 0.0002) whereas the *Vglut2* ^f/f^;Trpv1-Cre mice did not develop heat hyperalgesia; 15.4±1.0 s vs. 13.7±1.4 s (p = 0.96, power of test = 0.8)(Fig. 3C-D). We have previously shown that VGLUT2-mediated transmission from Trpv1-Cre neurons is responsible for acute heat transmission [10] and heat hyperalgesia associated with inflammation [13] and our novel finding indicates that VGLUT2-mediated glutamatergic transmission from Trpv1-Cre neurons is also responsible for mediating heat hyperalgesia associated with peripheral nerve injury. We observed a difference in baseline values between the control mice of each transgenic line (p = 0.0006, Mann-Whitney two-tailed test; Fig. 3A, C), which might be a result of background strain variations that has been shown to contribute to noxious heat sensitivity [26].

During embryonic development, TRPV1 is expressed in most cells that also express the cold receptor TRPM8 [3] resulting in Trpv1-Cre expression in a large part of *Trpm8*-expressing cells (65%) [25]. Since TRPM8 is known to be involved in cold (cooling) transmission [27, 28], cold hypersensitivity after peripheral inflammation via the Trpv1-Cre population [3] and cold allodynia associated with nerve injury [29], we set out to analyze the development of cold hypersensitivity after nerve injury in mice lacking the Trpv1-Cre population. Both control and *R26* ^DTA/wt^; Trpv1-Cre mice developed cold hyperalgesia; 0.67±0.21 s vs. 8.0±0.9 s (p<0.0001) and 1.0±0.35 s vs 4.7 ±0.5 s (p = 0.0002), respectively, albeit to a lesser extent in *R26* ^DTA/wt^; Trpv1-Cre mice (p = 0.02) (Fig. 3E-F), which could be the result of the extensive overlap between *Trpm8* cells and Trpv1-Cre activity *Vglut2* ^f/f^;Trpv1-Cre mice and controls both developed cold hyperalgesia to a similar extent (p = 0.48, power of test = 0.82); 0.89±0.28 s vs. 7.9±1.5 s (p = 0.004) and 0.63±0.19 s vs. 3.6±0.8 s (p = 0.0027), respectively (Fig. 3G-H), indicating that VGLUT2 is not involved in cold transmission in Trpv1-Cre neurons.

In summary, Trpv1-Cre neurons mediate heat hyperalgesia following peripheral nerve injury through VGLUT2-mediated glutamatergic signaling. Cold hypersensitivity, however, does neither depend fully on the Trpv1-Cre population nor on VGLUT2-mediated transmission from Trpv1-Cre neurons.

### VGLUT2-mediated signaling contributes to punctuate hyperalgesia via the Trpv1-Cre population

**Figure 4:**
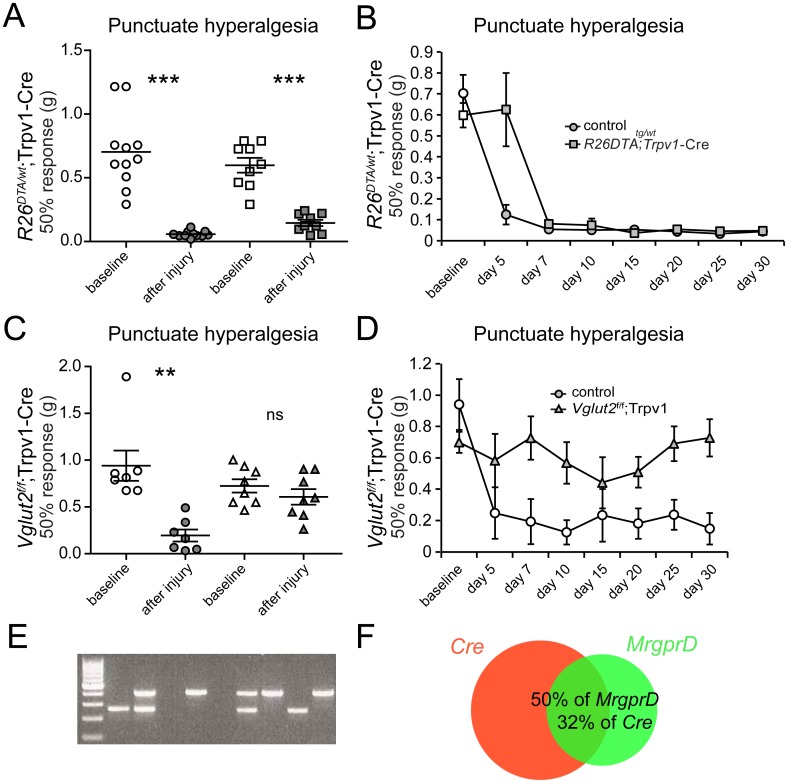
Glutamate mediates punctuate hyperalgesia associated with peripheral nerve injury via the Trpv1-Cre population. **(A-B)** Both control (n = 11) and *R26* ^DTA/w*t*^;Trpv1-Cre (n = 9) mice develop punctuate hyperalgesia but transgenes to a lesser extent. **(C-D)** Controls but not *Vglut2* ^f/f^;Trpv1-Cre mice develop punctuate hyperalgesia. Baseline (white) = response before PSNL, after injury (grey) = average response after PSNL. **(E)** Single-cell PCR analysis showed that out of 68 cells analyzed, 13 was positive for only *MrgprD* mRNA, 23 only for *Trpv1-Cre* mRNA, 11 expressed both markers, and 19 cells neither of the two markers. *Cre*-405 bp, *MrgprD*-271 bp. **(F)** The overlapping population corresponds to 50% of the MrgprD population and 32% of Trpv1-Cre population. Circles denotes controls, squares display *R26* ^DTA/w*t*^;Trpv1-Cre and triangles show *Vglut2* ^f/f^;Trpv1-Cre mice. **p<0.01, *** p<0.001, ns p>0.05, Mann-Whitney two-tailed test.

To evaluate the behavioral response to mechanical stimuli after nerve injury we used von Frey filaments in injured *R26* ^DTA/wt^; Trpv1-Cre mice and *Vglut2* ^f/f^;Trpv1-Cre mice. Control mice and *R26* ^DTA/wt^; Trpv1-Cre mice both developed punctuate hyperalgesia 0.70±0.09 g vs. 0.06±0.007 g (p<0.0001) and 0.60±0.06 g vs. 0.15±0.02 g (p = 0.0004) (Fig. 4A-B), respectively, which is consistent with the findings of Mishra et al [3]. However, the level of hyperalgesia was less pronounced in the *R26* ^DTA/wt^; Trpv1-Cre mice (p = 0.01), indicating that the Trpv1-Cre population do contribute to punctuate hyperalgesia.

Surprisingly, when analyzing *Vglut2* ^f/f^;Trpv1-Cre mice, we observed that control mice developed punctuate hyperalgesia 0.94±0.16 vs. 0.20±0.06 g (p = 0.002) whereas *Vglut2* ^f/f^;Trpv1-Cre mice did not. *Vglut2* ^f/f^;Trpv1-Cre mice responded at 0.72±0.07 g vs. 0.61±0.08 g (p = 0.32, power of test = 0.99), suggesting that VGLUT2-mediated signaling in Trpv1-Cre expressing neurons transmit punctuate hyperalgesia associated with nerve injury (Fig. 4C-D). This finding prompted us to investigate the overlap between the Trpv1-Cre population and the MrgprD population which previously been associated with mechanical sensation [30]. Our single cell analysis showed that 32% of the *Trpv1-Cre* population also expressed *MrgprD* which corresponded to 50% of the *MrgprD* expressing population (Fig. 4E-F). Interestingly, MrgprD has previously been shown to have little overlap with TRPV1 immunoreactivity [31, 32], which indicates that the observed behavioral phenotype might originate from Trpv1-Cre positive/MrgprD positive/TRPV1 negative neurons. Notably, the baseline response of the *Vglut2* ^f/f^;Trpv1-Cre mice is consistent with our previous findings [10].

In summary, VGLUT2-mediated glutamatergic transmission in Trpv1-Cre neurons is crucial for the development of heat and punctuate, but not cold, hyperalgesia following nerve injury.

## Discussion

Here we investigated the contribution of VGLUT2-mediated transmission in Trpv1-Cre neurons to the development of hyperalgesia after nerve injury. We show for the first time that a specific deletion of *Vglut*2 from Trpv1-Cre primary neurons resulted in decreased development of heat hyperalgesia as well as punctuate hyperalgesia after nerve injury, suggesting a central role for VGLUT2-mediated transmission in Trpv1-Cre neurons in the development of these pain states. Further, we show that removal of *Vglut2* does not affect the development of cold allodynia associated with nerve injury, indicating that VGLUT2-mediated transmission in Trpv1-Cre fibers is selective for heat and punctuate hyperalgesia.

### VGLUT2 and glutamate mediate nerve-injury associated heat hyperalgesia through the Trpv1-Cre population

Peripheral mechanisms of heat hyperalgesia, one of the frequent symptoms of neuropathy, have been reviewed extensively. However, the molecular basis of these processes and the role of the specific neuronal population in neuropathic pain have not been fully characterized. Despite the first reports discarding the involvement of TRPV1 in nerve injury associated heat hyperalgesia [1], more recent reports have reconsidered this hypothesis [33, 34, 35]. Glutamatergic transmission in the spinal cord has been related to long-term changes in transmission, leading to chronic pain. Especially NMDA-mediated signalling has been implicated in development of hyperalgesia and allodynia (reviewed by [36, 37]. Similarly, intrathecal injections of ketamine or dextrorphan, two non-competitive NMDA receptor antagonists have showed to reduce thermal hyperalgesia in rats [38]. It has however been difficult to separate the peripheral component of glutamatergic signalling in neuropathic conditions from the role of glutamatergic signalling in spinal cord interneurons. Recently, we and others showed that removal of peripheral VGLUT2-mediated transmission give rise to mice resistant to the development of heat hyperalgesia following nerve injury [14, 15]. We can now show that the Trpv1-Cre population of primary afferents is responsible for this phenotype.

### VGLUT2 and glutamate mediate nerve-injury associated punctuate hyperalgesia through the Trpv1-Cre population

The role of TRPV1 in punctuate hyperalgesia following nerve injury is controversial. RNA interference studies of the TRPV1 receptor prevent the development of punctuate hyperalgesia [39]. Also, intrathecal injections of TRPV1 specific antagonists has been shown to attenuate mechanical hypersensitivity associated with nerve injury [40]. On the contrary, TRPV1 knockout mice developed punctuate hyperalgesia comparable to control mice after nerve injury [39] and mice ablated of Trpv1-Cre neurons also show no significant differences in the degree of mechanical hypersensitivity after nerve injury compared to control mice [3]. Hence, while acute blockade of TRPV1 affects punctuate hyperalgesia after nerve injury, genetic ablation does not, which might indicate that developmental changes could compensate for the loss of TRPV1 or the TRPV1 population of neurons. Our analysis of the Trpv1-Cre population, using the diphtheria toxin line, can confirm that the population is partly redundant for punctuate hyperalgesia when ablated during development. However, mice ablated of Trpv1-Cre neurons do show a less pronounced state of punctuate hyperalgesia compared to control mice, which indicates that the population do contribute to punctuate hyperalgesia after nerve injury. Also, somewhat paradoxical, punctuate hyperalgesia after nerve injury was significantly attenuated in *Vglut2^f/f^;Trpv1-Cre* mice, indicating that the population, when injured, contribute to neuropathic states of pain via VGLUT2. This paradox could be the result of developmental compensations in the DRG due to the ablation of Trpv1-Cre neurons initiated when the Trpv1-Cre promoter is activated at approximately E13.5. Changed neuronal composition of the DRGs may result in changes during the waves of the genetic cascades leading to the differentiation of DRG neurons [41]. Hence, when the Trpv1-Cre population is absent, other populations important for punctuate hyperalgesia might compensate for this modification but when the population is still there, however with disturbed glutamatergic transmission, the plastic changes crucial for hypersensitivity are modulated, leading to decreased hypersensitivity. Our single cell analysis show that *Trpv1-Cre* mRNA expressing neurons also partially express *MrgprD* mRNA, a receptor that has been associated with mechanical sensation [30] in mainly non-peptidergic neurons [31, 32] which could indicate that the observed phenotype in *Vglut2^f/f^;Trpv1-Cre* mice might originate from non peptidergic MrgprD, Trpv1-Cre, VGLUT2 positive/TRPV1 negative primary afferents. This finding prompt for targeted *Vglut2* removal from the MrgprD population to investigate if MrgprD neurons are responsible for mediating punctuate hyperalgesia after nerve injury and if VGLUT2-mediated glutamatergic transmission is responsible for the signalling.

The role of glutamatergic transmission from primary afferent neurons in punctuate hyperalgesia after nerve injury is consistent with studies where all primary afferents or the Nav1.8 (Voltage gated sodium channel 1.8) population was ablated of VGLUT2 using a BAC generated mouse line [11, 15]. However, removal of *Vglut2* from the Nav1.8 population, using a knock-in Cre line, or from the peripherin population, which is suggested to incorporated most nociceptors, does not result in this phenotype [12, 14]. Collectively, this might indicate that VGLUT2 contributes to punctuate hyperalgesia in a specific subpopulation of primary afferent neurons and that loss of VGLUT2 can be compensated for by other signaling pathways The role of glutamate in the development of mechanical hypersensitivity has previously been shown using conventional VGLUT3 knockout mice which display decreased punctuate hyperalgesia after nerve injury [42].

In summary, these data strongly suggest a role for peripheral glutamate in the development of hyperalgesia after nerve injury. We thus propose that VGLUT2-mediated transmission in Trpv1-Cre neurons is an essential step in the cascade of plastic changes leading to sensitization after nerve injury. Future identification of peripheral components of the VGLUT2-dependent signalling cascade may therefore become therapeutic targets for the treatment of chronic states of pain.

## Supporting Information

S1 Fig Immunoreactivity towards red fluorescent protein (RFP) produced in Trpv1-Cre active neurons by the tdTomato line could be observed in dorsal root ganglia whereas no immunoreactivity towards RFP could be detected in abdominal tissue. Scale bars = 37 μm (A), 100 μm (B).
(DOCX)
