# Supplementary material for: VGLUT2 controls heat and punctuate hyperalgesia associated with nerve injury via TRPV1-Cre primary afferents
Source: PLoS One. 2015 Jan 23;10(1):e0116568. doi: 10.1371/journal.pone.0116568 (PMC4304805; doi:10.1371/journal.pone.0116568)
Supplement: S1 Fig — Scale bars = 37 μm (A), 100 μm (B). (DOCX) [file pone.0116568.s001.docx]

Supplementary figure 1


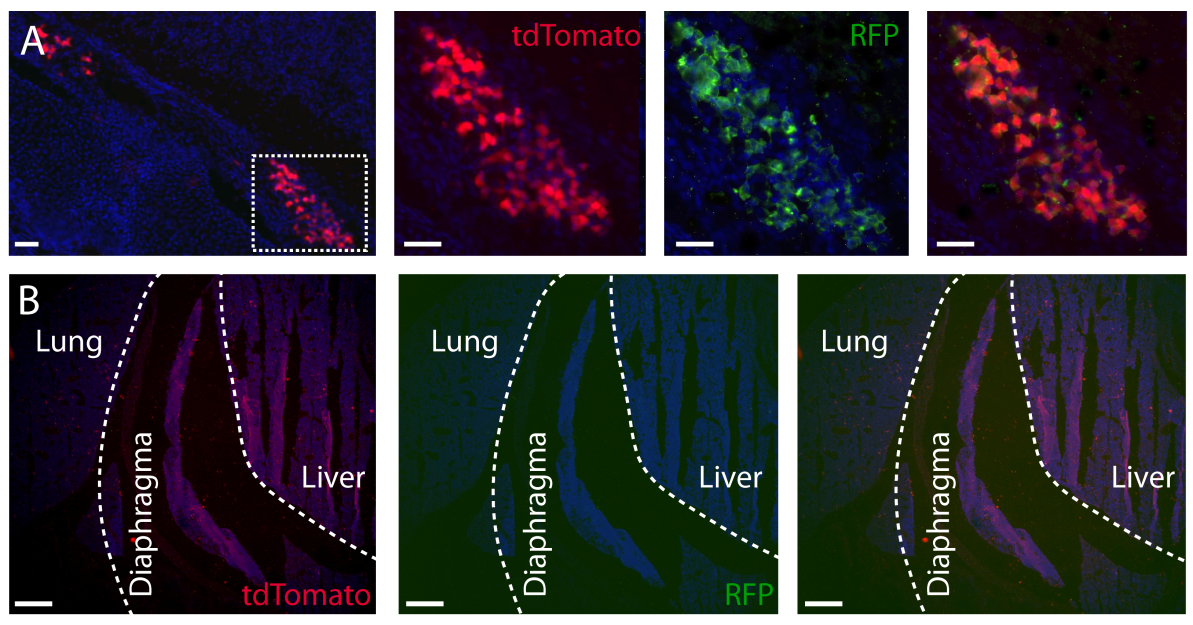


**Supplementary figure 1.** Immunoreactivity towards red fluorescent protein (RFP) produced in Trpv1-Cre active neurons by the tdTomato line could be observed in dorsal root ganglia whereas no immunoreactivity towards RFP could be detected in abdominal tissue. Scale bars=37 μm (A), 100 μm (B).
